# Roles of reactive oxygen species, mitochondrial membrane potential, and p53 in evodiamine-induced apoptosis and G2/M arrest of human anaplastic thyroid carcinoma cells

**DOI:** 10.1186/s13020-021-00505-3

**Published:** 2021-12-09

**Authors:** Chih-Chiang Chien, Ming-Shun Wu, Shih-Wei Chou, Ganbolor Jargalsaikhan, Yen-Chou Chen

**Affiliations:** 1 Department of Nephrology, Chi-Mei Medical Center, Tainan, Taiwan; 2 Department of Food Nutrition, Chung Hwa University of Medical Technology, Tainan, Taiwan; 3 Division of Gastroenterology, Department of Internal Medicine, Wan Fang Hospital, Taipei Medical University, Taipei, Taiwan; 4 Division of Gastroenterology and Hepatology, Department of Internal Medicine, School of Medicine, College of Medicine, Taipei Medical University, Taipei, Taiwan; 5 Graduate Institute of Medical Sciences, College of Medicine, Taipei Medical University, 250 Wu-Hsing St, 11031 Taipei, Taiwan; 6 International MS/PhD Program in Medicine, College of Medicine, Taipei Medical University, 11031 Taipei, Taiwan; 7 Liver Center, 14230 Ulaanbaatar, Mongolia; 8 Cancer Research Center and Orthopedics Research Center, Taipei Medical University Hospital, Taipei, Taiwan; 9 Cell Physiology and Molecular Image Research Center, Wan Fang Hospital, Taipei Medical University, Taipei, Taiwan

**Keywords:** Anaplastic thyroid cancer (ATC), Reactive oxygen species (ROS), Mitochondria membrane potential (MMP), Evodiamine (EVO), Apoptosis, G_2_/M arrest

## Abstract

**Background:**

Our previous studies have shown that evodiamine (EVO) as paclitaxel and nocodazole could trigger apoptosis in various human cancer cells including human renal cell carcinoma cells, colorectal carcinoma cells, and glioblastoma cells. This study aims to investigate the anti-cancer effects of EVO on human anaplastic thyroid carcinoma (ATC) cells, and underlining mechanism.

**Methods:**

Two different endogenous p53 status human anaplastic thyroid carcinoma (ATC) cells including SW1736 (wtp53) and KAT4B (mutp53) were applied in the present study. The cytotoxicity of EVO on ATC cells was measured by MTT assay, and apoptosis and G2/M arrest were detected by propidium iodide (PI) staining followed by flow cytometry. Expression of indicated proteins was evaluated by Western blotting analysis, and pharmacological studies using chemical inhibitors and siRNA were performed for elucidating underlying mechanism. The roles of mitochondrial membrane potential and reactive oxygen species were investigated by flow cytometry using DiOC6 and DCFH-DA dye, respectively.

**Results:**

SW1736 (wtp53) cells showed a higher apoptotic percentage than KAT4B (mutp53) cells in response to EVO stimulation via a flow cytometric analysis. Mechanistic studies showed that increased p53 and its downstream proteins, and disrupted MMP with increased intracellular peroxide production participated in EVO-induced apoptosis and G2/M arrest of SW1736 cells. In EVO-treated KAT4B cells, significant increases in G2/M percentage but little apoptotic events by EVO was observed. Structure-activity analysis showed that an alkyl group at position 14 was critical for induction of apoptosis related to ROS production and MMP disruption in SW1736 cells.

**Conclusion:**

Evidence indicated that the endogenous p53 status affected the sensitivity of ATC cells to EVO-induced apoptosis and G2/M arrest, revealing the potential role of p53 related to increased ROS production and disrupted MMP in the anticancer actions of EVO, and alkylation at position 14 of EVO is a critical substitution for apoptosis of ATC cells.

## Background

Three types of human thyroid cancers including differentiated thyroid cancer (DTC), poorly DTC (PDTC), and anaplastic thyroid cancer (ATC) have been identified as endocrine malignancy [1], and ATC is rare among human thyroid cancers with aggressive phenotype and a poor prognosis, and insensitive to available current chemotherapeutic agents and radiotherapy [2, 3]. Due to no effective treatments for ATC that has reached an advanced or recurrent level, there is an urgent need for a novel, effective agent for treating ATC.

Apoptosis is a process of programmed cell death in maintaining cell homeostasis, and it has been shown that avoidance of apoptosis is a cause of cancer [4, 5]. There are several apoptotic characteristics such as specific morphological and biochemical characteristics including membrane blebbing, DNA fragmentation, mitochondrial depolarization, and caspase activation were observed in cells undergoing apoptosis [6, 7]. Therefore, agents with activity that induces apoptosis to kill or overcome oncogenic resistance have potential to be developed for cancer patients. In addition to apoptosis, abnormal cell cycle events take place in cancer cells, and defective cell cycle progression results in unlimited cell proliferation of cancers [8, 9]. Clinical anticancer drugs such as paclitaxel and nocodazole kill cancer cells via arrest of the cell cycle at specific phases such as the G2/M phase leading to apoptosis [10, 11]. Both Cyclin-dependent protein kinases (CDKs) and D-type cyclins control cell cycle progression at specific cell cycle checkpoints, indicating to be molecular targets for cancer treatment [12, 13]. The tumor suppressor, p53, is a vital guardian of cellular genomes, and plays a critical role in cellular processes including apoptosis, cell cycle arrest, and cell senescence [14]. It has been shown that p53 inhibits the survival of cancer cells by triggering the release of proapoptotic factors leading to apoptosis [15]. Additionally, decreased p53 functions suppress apoptosis and contribute to chemotherapeutic resistance of cancer cells [16]. Phosphorylation of the p53 protein was reported in several previous studies, and increased phosphorylation of the p53 protein was suggested to induce apoptosis [15, 17]. Previous papers indicated the role of p53 in regulating apoptosis and cell cycle progression which was related to the sensitivity of cancer cells to chemotherapy [18, 19].

Naturally derived compounds are important as therapeutic leads, and more than half of cancer therapeutic agents are derived from natural chemicals and their derivatives. There are several anticancer agents, such as vincristine and paclitaxel, derived from plants that have been used to clinically treat various human cancers [20]. Evodiamine (EVO) is a compound isolated from *Evodia rutaecarpa*, and various biological effects by EVO including antioxidant, anti-inflammatory, anti-neuronal injury, anti-renal injury, antitumor, anti-allergic, and antiviral properties have been found [21–23]. Our recent studies demonstrated that EVO was able to inhibit the proliferation of human renal cancer cells, glioblastoma cells, and colorectal carcinoma cells, and activation of protein kinase RNA (PKR)-like endoplasmic reticulum (ER) kinase (PERK), and c-Jun N-terminal kinase (JNK) is identified [24–26]. Although EVO’s inhibition of tumor growth was reported, actions of EVO on the viability and cell cycle progression of ATC cells and their relationship with the endogenous p53 status are still unclear.

In this study, two ATC cells including SW1736 and KAT4B were used in the study, and the levels of p53 protein were highest in KAT4B cells, and very low to undetectable levels in SW1736 cells. The sequence analysis revealed mutant p53 (mutp53) status with a substitution in codon 273 in KAT4B, and no p53 mutation was identified in RNA from SW1736, indicating to be wild type p53 (wtp53) [27]. We found that EVO could inhibit cell proliferation and increase apoptosis and G2/M arrest in ATC SW1736 (wtp53) and KAT4B (mutp53) cells. SW1736 cells showed significantly higher sensitivity to EVO’s actions than KAT4B cells. Analysis of structural significance of EVO on apoptosis and the critical role of the endogenous p53 status in the sensitivity of ATC cells to EVO-induced apoptosis and G2/M arrest related to mitochondrial membrane potential and ROS production were demonstrated herein.

## Methods

### Cell culture

The human anaplastic thyroid cancer cell line, SW1736 and KAT4B, were obtained from American Type Culture Collection (Manassas, VA, USA). The cells were cultured in Roswell Park Memorial Institute (RPMI) 1640 Medium (Thermo Fisher Scientific Inc) supplemented with 10% heat-inactivated fetal bovine serum (FBS; Gibco/BRL, Grand Island, NY, USA), antibiotics (100 U/mL penicillin A and 100 U/mL streptomycin), and incubated in a humidified atmosphere containing 5% CO_2_ at 37 °C.

### Agents

The chemical reagents of EVO, pifithrin-α, 5-bromo-4-chloro-3-indolyl phosphate (BCIP), 3-(4,5-dimethylthiazol)-2-yl-2,5-diphenyltetrazolium bromide (MTT), paclitaxel, nocodazole, nitro blue tetrazolium (NBT), were obtained from Sigma Chemical (St. Louis, MO, USA). Antibodies of cdc25c (55 kDa; Catalog no. sc-13,138), cdc2 (34 kDa; Catalog no. sc-8395), p53 (53 kDa; Catalog no. sc-126), p27 (27 kDa; Catalog no. sc-1641), p21 (21 kDa; Catalog no. sc-6246), α-tubulin (α-TUB; 55 kDa; Catalog no. sc-5286), and GAPDH (37 kDa; Catalog no. sc-47,724) were obtained from Santa Cruz Biotechnology (Santa Cruz, CA, USA). Antibodies against poly(ADP ribose) polymerase (PARP; 116 kDa; Cleaved fragment 89 KDa; Catalog no. #9542), caspase-3 (35 kDa; Cleaved fragments 17/19 kDa; Catalog no. #14,220), and phosphorylated (p)-p53 (53 kDa; Catalog no. #9284; Ser 15) were obtained from Cell Signaling Technology (Beverly, MA, USA). Other chemicals not mentioned above were obtained from Sigma Chemical.

### MTT assay

EVO was tested on SW1736 and KAT4B ATC cell lines using an MTT assay based on the ability of live cells may convert MTT to dark blue formazan. Both cells grown in DMEM-10 % FBS were plated at a density of 10^5^ cells/well in 24-well plates, and the cells were incubated with medium alone or medium containing various concentrations of EVO diluted in DMSO. After incubation for different times, the number of metabolically active cells were determined by MTT assay. Briefly, the supernatant was removed, and 30 µl of the tetrazolium compound, MTT, and 270 µl of fresh DMEM-10% FBS medium were added at a final MTT concentration 0.5 mg/l. After incubation for 4 h at 37 °C, 200 µl of 0.1 N HCl in 2-propanol was placed in each well to dissolve the tetrazolium crystals. Finally, the absorbance at a wavelength of 600 nm was recorded using an enzyme-linked immunosorbent assay (ELISA) plate reader (MR-5000; Dynatech Laboratories Inc., Chantilly, VA).

### In vitro morphology

Human ATC cells grown at a density of 10^5^ cells/well in 24-well plates for 24 h followed by treatment with various components for an additional 24 h. Cells were fixed with 3.7% formaldehyde followed by Giemsa staining for 10 min. The morphological changes of both ATC cells under different treatments were observed under a light microscope. Giemsa is able to bind with chromosomes, and apoptotic cells characterized by nuclear condensation (dark) were observed microscopically.

### DNA fragmentation analysis

The phenol/chloroform/isoamyl alcohol procedure was used to extract DNA from aliquots of cell lysates (5 × 10^6^ cells per sample) that had been digested with proteinase. The DNA was ethanol-precipitated, dissolved in Tris-EDTA buffer, incubated with RNase A (50 µg/ml) for 30 min at 37 °C, and then analyzed by electrophoresis in 1.5% agarose gels.

### Western blotting

Human ATC cells treated with different components for different times, and cellular extracts were collected with 100 µl cell lysis buffer (50 mM Tris (pH 8.0), 150 mM NaCl, 1% Nonidet P-40, 0.5% sodium deoxycholate, 0.1% sodiumdodecyl sulfate). The protein concentration was determined by protein assay kit (Bio-Rad), and the cellular extracts (30 µg) were subjected to 8% sodium dodecylsulfate (SDS)-polyacrylamide mini gels for PARP detection and 12% SDS-polyacrylamide minigels for detecting indicated proteins on a PVDF membrane (Millipore, Bedford, MA, USA). Membranes were incubated at 4˚C with 1% bovine serum albumin (BSA) and then incubated with the indicated primary antibodies (1:1000) for a further 3 h at room temperature followed by incubation with an alkaline phosphatase-conjugated immunoglobulin G (IgG) antibody for 1 h. Proteins were visualized by incubating with the colorimetric substrates, NBT chloride and BCIP.

### Measurement of the mitochondrial membrane potential (MMP)

SW1736 and KAT4B cells were incubated with different concentrations of EVO for 12 h followed by staining with 40 nM DiOC6(3) for 15 min at 37 °C. After washed with ice-cold PBS, cells were collected by centrifugation at 500 × *g* for 10 min, and resuspended in 500 ml of PBS for flow cytometric analysis of fluorescence intensities of DiOC6(3) (FACScan, Becton Dickinson) in cells using respective excitation and emission settings of 484 and 500 nm, and at least ten thousand cells were analyzed for each data point.

### Measurement of ROS generation

ROS generation by EVO was assayed by using a cell permeable fluorescent indicator 2,7-dichlorodihydrofluorescein-diacetate (DCFH-DA). DCFH-DA is hydrolyzed to the non-fluorescent polar derivative, DCFH, within cells, and the oxidized to highly fluorescent by ROS. SW1736 and KAT4B cells treated by different concentrations of EVO were incubated in the dark for 10 min at 37 °C with 50 µmol/l DCFH-DA for another 30 min. DCF fluorescence intensities were measured using a flow cytometer (FACScan, Becton Dickinson) with excitation at 488 nm and emission at 530 nm. The values were described as % relative fluorescent intensity compared to the control.

### Annexin V-FITC/PI staining

SW1736 cells with or without EVO treatment for different times were harvested with trypsin-EDTA, washed, and exposed to Annexin V-FITC (Roche Diagnostics, Laval, QC, Canada) diluted according to manufacturer’s protocol and 1 µg/ml PI in detection buffer (10 mM HEPES, 10 mM NaCl, and 5 mM CaCl_2_) for 15 min at room temperature. Cells were analyzed on FL1 and FL2 with appropriate controls using a FACSCaliburflow cytometer and CellQuest softwar.

### Detection of cell cycle progression and hypodiploid cells by EVO in cells

To determine whether EVO could induce cell cycle arrest and apoptosis in human ATC cells, SW1736 and KAT4B cells were plated in 24-well plates in duplicate for 24 h, and treating with complete medium (10% FBS) containing different concentrations of EVO for 24 h. Cells were harvested by exposing cells to a 0.25% trypsin-EDTA solution for 10 min, then centrifuged, washed in PBS, and fixed in 3 mL ice-cold 100% ethanol. Cells were stored in ethanol for at least 24 h at -20 °C, then collecting the cell pellets and resuspended in PBS containing 0.1 % v/v Triton X-100, 0.2 mg/ml DNase-free RNase A, and 20 µg/ml PI. All samples were incubated at room temperature for 30 min. The cell cycle distribution and percentage of hypodiploid cells of SW1736 and KAT4B cells were determined by FACScan Flow Cytometry using a 488 nm argon (FACScan, Becton Dickinson) [28]. Due to Nuclear DNA content is lost during apoptosis, a hypodiploid (or sub-G1) DNA profile can be detected by flow cytometry analysis using PI staining. Therefore, the presence of cells with DNA stainability lower than that of G1-cells (hypodiploid cells) has been measured as percentage of hypodiploid cells in ATC cells after various treatments here.

### Introducing p53 and scramble siRNA into SW1736 and AKT4B cells

P53 small interfering RNA (siRNA) (Santa Cruz Biotechnology; sc-29,435) and scrambled (control; SC) siRNA (Santa Cruz Biotechnology; sc-37,007) of 5 µg was added to 200 µl medium without serum for 5 min, and LipofectAMINE reagent (Life Technologies, Grand Island, NY, USA) of 5 µl was added to 200 µl medium without serum for 5 min, respectively. Then, the above two solutions mixed at room temperature for 30 min are ready for transfection experiments, and SW1736 and KAT4B cells were incubated with the transfection complex solutions containing p53 siRNA or SC siRNA at 37 °C for 8 h, and then re-incubate in complete medium for an additional 24 h for experimental needs. SC siRNA, a nontargeting siRNA, designed as a negative control in the study. The efficiency of siRNA knockdown was determined by Western blotting for p53 protein detection.

### Statistical analysis

Data obtained from three independent experiments were expressed as the mean ± standard deviation (SD). The significance of the difference from the respective controls for each experimental or between indicated groups was assayed using a one-way analysis of variance (ANOVA) with a post-hoc Bonferroni analysis when applicable, and p values of < 0.05 (*) or < 0.01 (**) were considered statistically significant.

## Results

### EVO reduces the viability of SW1736 and KAT4B ATC cells with differential 50% inhibitory concentration (IC50) values

**Fig. 1:**
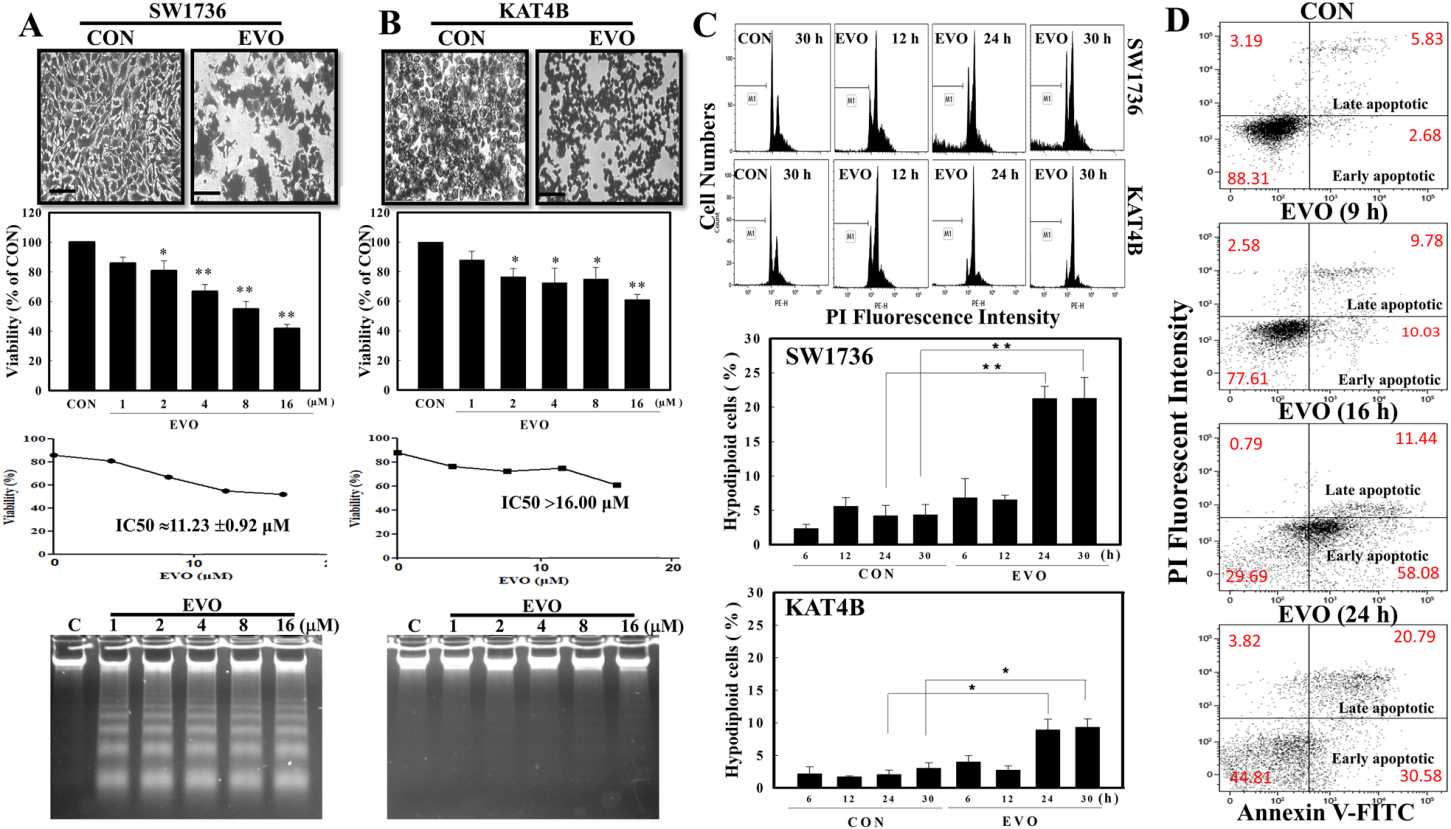
Effects of evodiamine (EVO) on the viability of SW1736 and KAT4B anaplastic thyroid cancer (ATC) cells. EVO reduced the viability with morphological changes and DNA ladders in SW1736 (**A**) and KAT4B (**B**) cells via MTT assay, Giemsa staining, and agarose electrophoresis, respectively. Both cell lines were treated with EVO (8 µM) for 24 h, and cellular morphology, viability, and DNA ladder was analyzed as described in Methods. Scale bar (), 100 μm. **C** EVO differentially induced hypodiploid cells in SW1736 and KAT4B cells. Both cell lines were treated with and without EVO (8 µM) for different times, and the ratio of hypodiploid cells was examined by a flow cytometric analysis using PI staining. (Upper panel) A representative of the flow cytometric analysis is shown. (Lower panel) Data of the hypodiploid cell ratio from three independent experiments were collected and statistically analyzed. Each data point was calculated from three triplicate groups, and data are shown as the mean ± SD. *p < 0.05 and **p < 0.01 denote a significant difference between the indicated groups. **D** EVO induced apoptosis in SW1736 cells on results from an Annexin V-PI binding assay via flow cytometric analysis. SW1736 cells were treated with EVO (8 µM) for 9, 16, and 24 h. Representative flow cytometry scatter plots depict percentage of AnnexinV staining after EVO treatment. Cells in lower right quadrant IV (Annexin V+/PI−) are in the early apoptotic and in upper right quadrant II (Annexin V+/PI+) are in the late apoptotic stage. Data were shown as mean from three-independent experiments

Firstly, we investigated if EVO exerts antitumor activity against SW1736 and KAT4B ATC cells. In the presence of EVO treatment, significant morphological changes with decreased cell numbers were detected in both cell lines under microscopic observations via Giemsa staining (Fig. 1A, B). EVO concentration-dependently reduced the viability of ATC cells according to an MTT assay, and IC50 values of EVO toward SW1736 and KAT4B cells were 11.2 and > 16 µM, respectively. Results of DNA integrity assay showed that DNA ladders were observed in EVO-treated SW1736, but not KAT4B, cells. Data of the flow cytometric analysis via propidium iodide (PI) staining showed that an increased ratio of hypodiploid cells by EVO was detected in SW1736 and KAT4B cells, and the ratio of those cells in SW1736 cells was significantly higher than that in KAT4B cells (Fig. 1C). A representative of flow cytometric data is shown in Fig. 1C (upper panel), and statistical analysis of the ratio of hypodiploid cells under EVO treatment for different times in both cell lines for different times is shown in Fig. 1C (lower panel). We further investigated the apoptotic effects of EVO in SW1736 cell using Annexin V-FITC assays. Annexin V/PI staining was performed to determine the percentages of early apoptotic and late apoptotic cells. As shown in Fig. 1D, treatment of SW1736 cells with EVO (8 µM) for 9, 16, and 24 h produced increases in the percentage of early and late apoptotic cells. (Fig. 1D).

### Differential Casp-3 activation, loss of mitochondrial membrane potential (MMP), and increased intracellular peroxide production by EVO in ATC cells

**Fig. 2:**
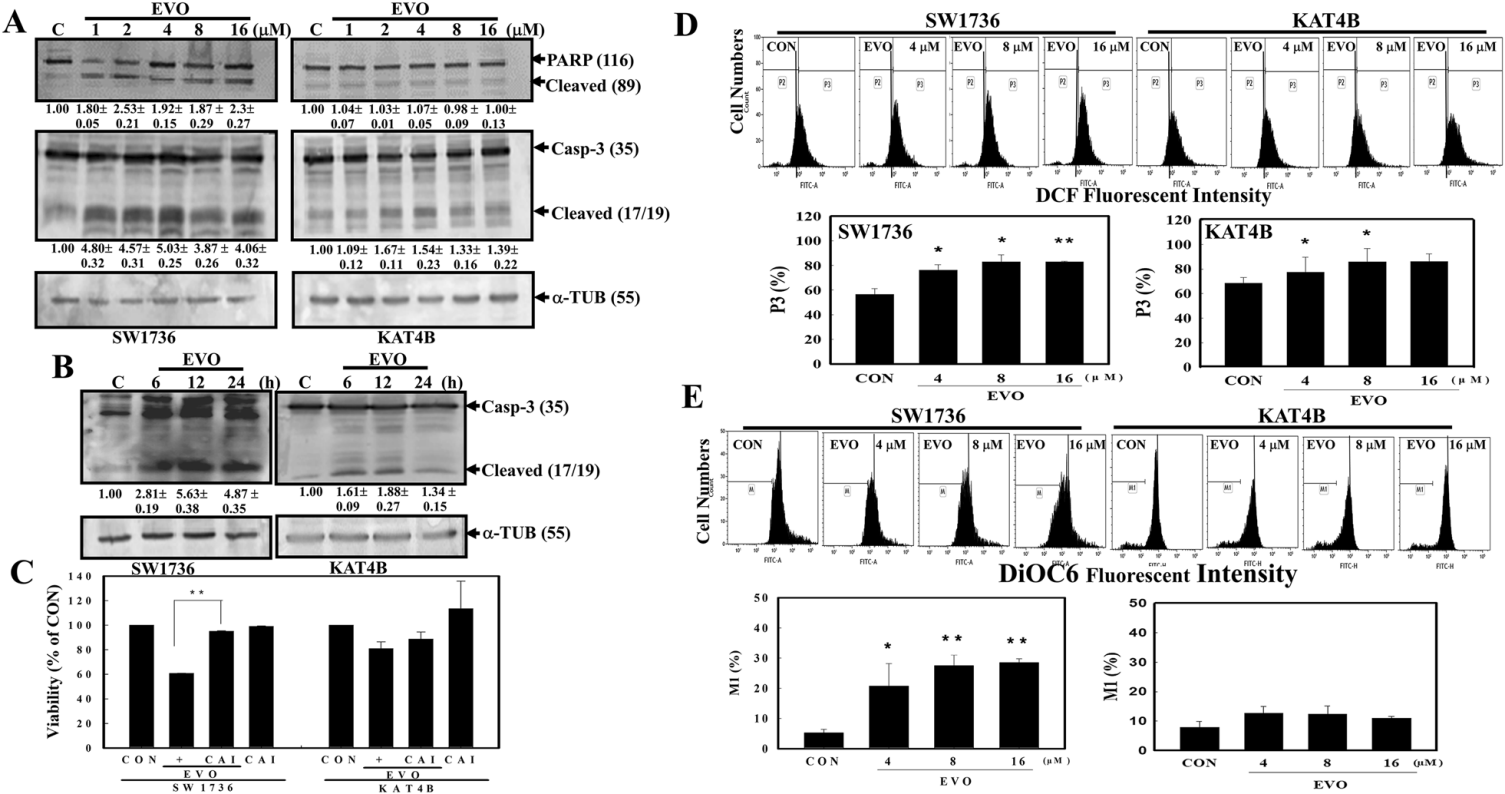
Effects of EVO on cleavages of poly(ADP ribose) polymerase (PARP) and caspase (Casp)-3 protein, mitochondrial membrane potential, and intracellular peroxide production in SW1736 and KAT4B cells. **A** EVO induced Casp-3 and PARP protein cleavages in SW1736 and KAT-4B cells in a concentration-dependent study. Both cell lines were treated with different concentrations (1, 2, 4, 8, and 16 µM) of EVO for 24 h, and expressions of PARP, Casp-3, and α-tubulin (α-TUB; as the internal control) were examined by Western blotting. **B** EVO induced Casp-3 protein cleavages in SW1736 and KAT-4B cells in a time-dependent study. Both cell lines were treated with EVO (8 µM) for different times (6, 12, and 24 h), and expressions of Casp-3, and α-tubulin (α-TUB; as the internal control) were examined by Western blotting. The intensity of each cleaved fragment in (**A**) and (**B**) was examined by a densitometric analysis (Image J) and expressed as multiples of the control. Each data point was calculated from triplicate groups, and data are shown as the mean ± SD. **C** Addition of a pan-caspase inhibitor (Ac-VAD-FMK; CAI) significantly inhibited EVO-induced cell death in SW1736 cells, but no significance in KAT4B cells. Both cell lines were incubated with a pan-caspase inhibitor (10 nM; CAI) for 4 h followed by EVO (8 µM) treatment for an additional 24 h. Viability of cells was examined by an MTT assay. **D** Alternative increment of intracellular peroxide level by EVO in SW1736 and KAT4B cells. Cells were treated with different concentrations of EVO (4, 8, and 16 µM) for 12 h, and intracellular peroxide levels were detected by a flow cytometric analysis using DCFH-DA as a fluorescent dye. **E** Disruption of the mitochondrial membrane potential (MMP) by EVO in anaplastic thyroid cancer (ATC) SW1736 and KAT4B cells. Cells were treated with different concentrations of EVO (4, 8, and 16 µM) for 12 h, and the MMP was detected by a flow cytometric analysis using DiOC6 as a fluorescent dye. (Upper panel) a representative of flow cytometric data was shown. (Lower panel) data from three-independent experiments was shown. Each data point was calculated from triplicate groups, and data are shown as the mean ± SD. *p < 0.05 and **p < 0.01 denote a significant difference compared to the control (CON)

Because Casp-3 is an executioner of apoptosis and PARP is a downstream protein of Casp-3, The expressions of caspase 3 and PARP proteins including intact and cleaved forms in SW1736 and KAT4B cells under EVO stimulation was detected here. Data of Western blotting indicated that EVO at different concentrations induced cleavage of Casp-3 and PARP proteins in SW1736 cells, but only slight increases were observed in KAT4B cells (Fig. 2A). In EVO treatment for different times, increased cleavage of Casp-3 were clearly observed in SW1736 cells, and less cleavage was detected in KAT4B cells (Fig. 2B). The addition of a pan caspase inhibitor (z-VAD-FMK, CAI) significantly reduced the viability of SW1736 cells by EVO (Fig. 2C). Analysis of intracellular peroxide level in both ATC cells by EVO treatment showed that increased intracellular peroxide production was significantly identified in EVO-treated SW1736 cells, but slightly increases were found in AKT4B cells (Fig. 2D). Furthermore, the mitochondrial membrane potentials (MMPs) of SW1736 and KAT4B cells with and without EVO stimulation were detected by a flow cytometric analysis using DiOC6 as a mitochondrial fluorescent probe for MMP detection. As shown in Fig. 2E, EVO concentration-dependently reduced MMPs in SW1736 cells but not in KAT4B cells.

### Increased G2/M arrest related to alternative expressions of cell cycle regulatory proteins by EVO in SW1736 and KAT4B cells

**Fig. 3:**
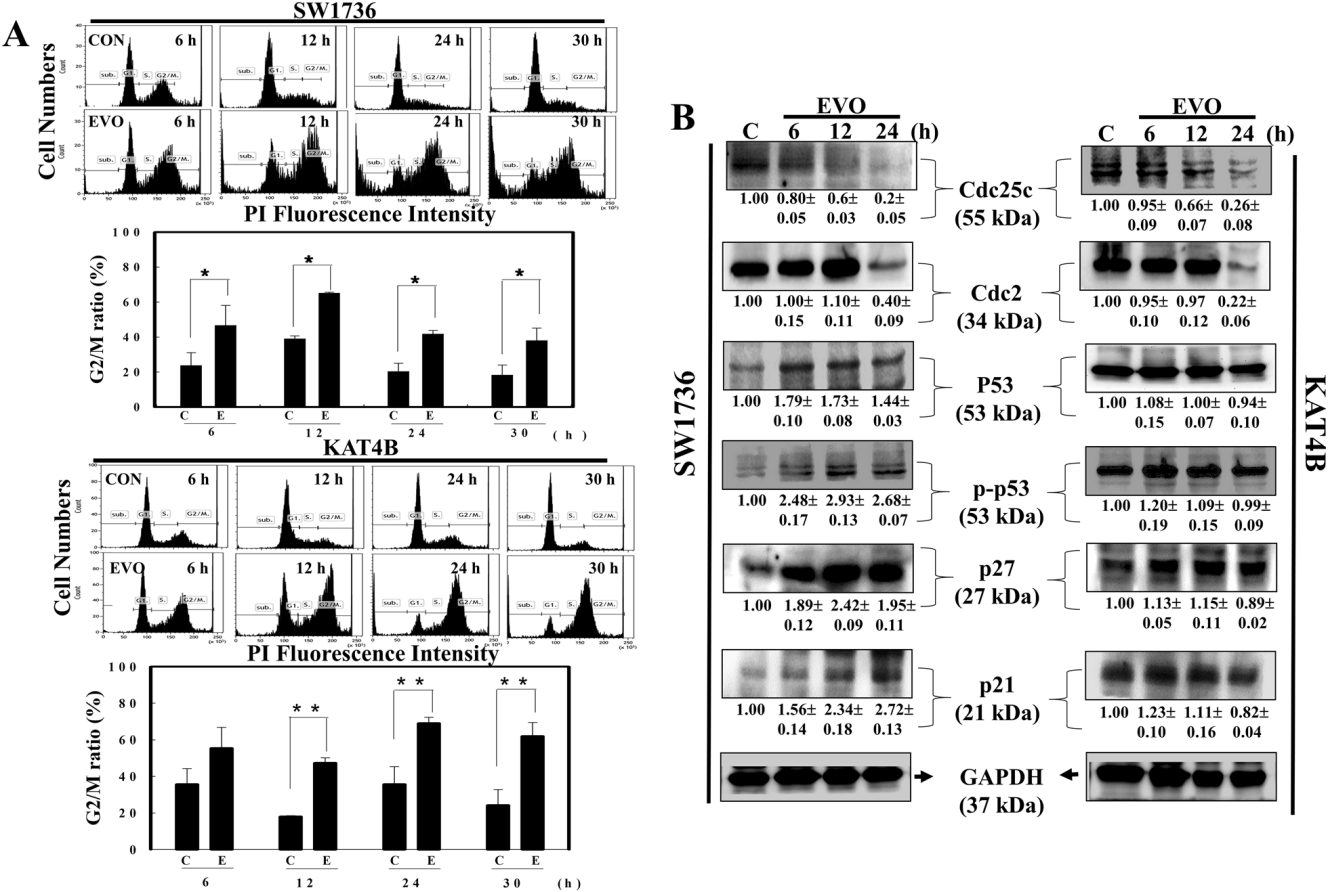
Increased G_2_/M ratio with alternative expression of indicated proteins by EVO in SW1736 and KAT4B cells. **A** Increased G_2_/M ratio by EVO in SW1736 (Upper panel) and KAT4B (Lower panel) cells. Both cell lines were treated with and without EVO (8 µM) for different times (6, 12, 24, and 30 h), and cell cycle progression was analyzed by flow cytometry via PI staining. A representative of the flow cytometric analysis was shown on the top of each panel; data were calculated from three triplicate groups and statistically analysis was shown below. **B** Alternative expressions of indicated proteins including p53, phosphorylated (p)-p53, cdc25c, cdc2, p27, and p21 proteins in SW1736 (left panel) and KAT3B cells (right panel) under EVO (8 µM) stimulation for different times (6, 12, and 24 h). Both cell lines were treated with EVO for different times, and expressions of indicated proteins were examined by Western blotting using specific antibodies. The intensity of each band was examined by a densitometric analysis (Image J), and results are expressed as multiples of the control. Each data point was calculated from triplicate groups, and data are shown as the mean ± SD. *p < 0.05 and **p < 0.01 denote a significant difference between indicated groups

The effect of EVO on the cell cycle progression of SW1736 and KAT4B ATC cells for different times (6, 12, 24, and 30 h), and percentages of cells at the G1 and G2/M phases were measured by a flow cytometric analysis via PI staining. As shown in Fig. 3A, an increased G2/M ratio of SW1736 cells was detected at various times after EVO stimulation with significant decreases in the G1 ratio (Fig. 3A; data not shown). In the same part of the experiment, EVO treatment induced increases in the G2/M ratio and decreases in the G1 ratio in KAT4B cells at 12, 24, and 30 h (Fig. 3A; data not shown). Expressions of cell cycle regulatory proteins, including cdc25c, cdc2, p53, p-p53, p27, p21, and GAPDH, were examined in EVO-treated SW1736 and KAT4B cells by Western blotting using specific antibodies. As illustrated in Fig. 3B, decreased cdc25c and cdc2 protein expressions by EVO were detected in both SW1736 and KAT4B cells. It was shown that SW1736 and KAT4B cells possess wild-type and mutated p53, respectively [27]. Under EVO stimulation, increased p53, p-p53, p27, and 21 protein expressions were detected in SW1736 cells, but not in KAT4B cells. This indicates that differential expression of p53 and its downstream proteins such as p27 and p21 might participate in alternative sensitivity of SW1736 and KAT4B cells to EVO-induced apoptosis.

### A chemical p53 inhibitor, pifithrin-α, inhibited EVO-induced apoptosis and G2/M arrest in SW1736 cells but not in KAT4B cells

**Fig. 4:**
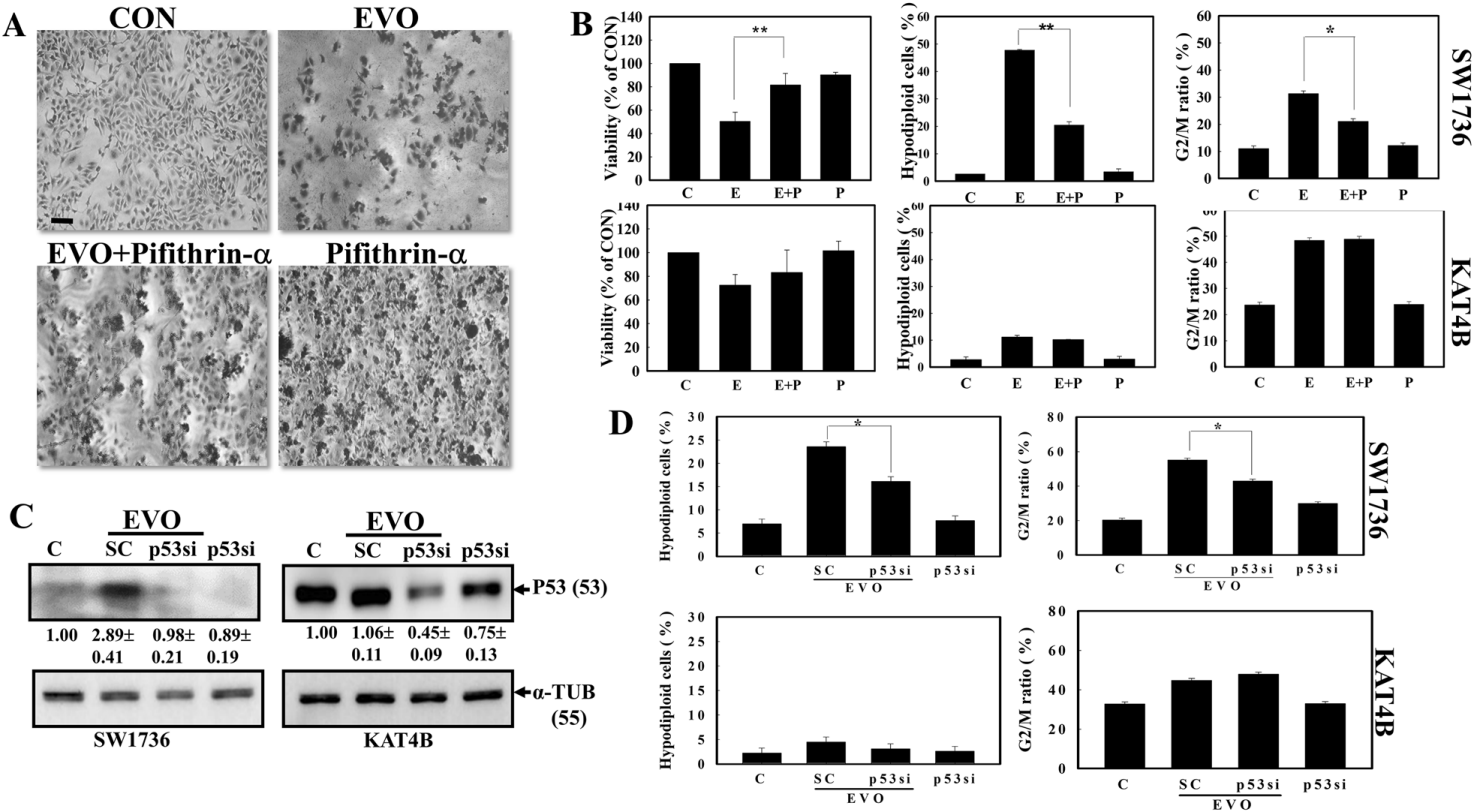
The p53 inhibitor, pifithrin-α and Knockdown of p53 protein expression by p53 siRNA, reversed the action of evodiamine (EVO) in SW1736 cells but not in KAT4B cells. **A** Morphological alterations in EVO-treated SW1736 cells after the addition of pifithrin-α. SW1736 cells were treated with pifithrin-α for 30 min followed by EVO (8 µM) stimulation for 24 h. The morphology of SW1736 cells was observed microscopically via Giemsa staining. Scale bar (), 100 μm (**B**) Pifithrin-α (P) reversed the cytotoxic effect of EVO (**E**) against the viability, hyperdiploid cells, and G2/M arrest of SW1736 cells but not KAT4B cells. Data are presented as the percentage of the control. **C** Introduction of p53 siRNA reduced endogenous p53 protein in both the SW1736 and KAT4B cell lines. Both cell lines were transfected with scrambled (SC) or p53 siRNA followed by EVO (8 µM) treatment for 24 h. Expression of p53 and internal control α-tubulin (TUB) protein was examined by Western blotting using specific antibodies. The intensity of each p53 band was examined by a densitometric analysis (Image J) and multiples of the control was calculated from triplicate groups as the mean ± SD. **D** p53 siRNA inhibited increases in the hypodiploid cell ratio and G2/M percentage in EVO-treated SW1736 cells but not KAT4B cells. As described in **A**, the ratio of hypodiploid cells and G2/M percentage were examined by a flow cytometric analysis. Each data point was calculated from triplicate groups, and data are shown as the mean ± SD. *p < 0.05 denotes a significant difference between indicated groups

In order to elucidate the role of p53 in EVO-induced apoptosis and G2/M arrest of ATC cells, pifithrin-α a reversible inhibitor of p53-mediated apoptosis and p53-dependent gene transcription such as p21 expression was applied in the present study [29]. As illustrated in Fig. 4A, decreased cell intensity by EVO was inhibited by pifithrin-α addition to SW1736 cells under microscopic observation via Giemsa staining. Data of the MTT assay showed that the addition of pifithrin-α reversed the EVO inhibition of viability of SW1736 cells but not KAT4B cells (Fig. 4B; Left panel). Additionally, increased ratios of hypodiploid cells and arrest at the G2/M phase by EVO in SW1736 cells were inhibited by adding pifithrin-α; however pifithrin-α did not affect EVO’s actions including hypodiploid cells and G2/M arrest in KAT4B cells (Fig. 4B; Middle and Right panel).

### Knockdown of p53 protein expression by transfection of p53 small interfering (si)RNA inhibited EVO actions in SW1736 cells but not in KAT4B cells

We further applied p53 siRNA to knock down endogenous p53 protein expression to study the role of p53 in EVO-induced cell death and G2/M arrest of ATC cells. As shown in Fig. 4C, an increased p53 protein level by EVO was determined in SW1736 cells, which was inhibited by p53 siRNA transfection. In KAT4B cells, a high endogenous level of the p53 protein was detected, EVO did not induce p53 protein expression as observed in Fig. 4C, and introducing p53 siRNA significantly reduced the endogenous p53 protein level. Analysis of the hypodiploid cell ratio by flow cytometry showed that p53 siRNA significantly reduced the increases in the hypodiploid cell ratio in EVO-treated SW1736 cells; however, no change was observed in KAT4B cells with or without introducing p53 siRNA (Fig. 4C; left panel). In the same part of the experiment, p53 siRNA significantly reduced the EVO-induced G2/M ratio in SW1736 cells but not in KAT4B cells according to a flow cytometric analysis (Fig. 4D; Right panel).

### Structure-activity analysis of EVO on apoptosis and p53 protein expression in SW1736 cells

**Fig. 5:**
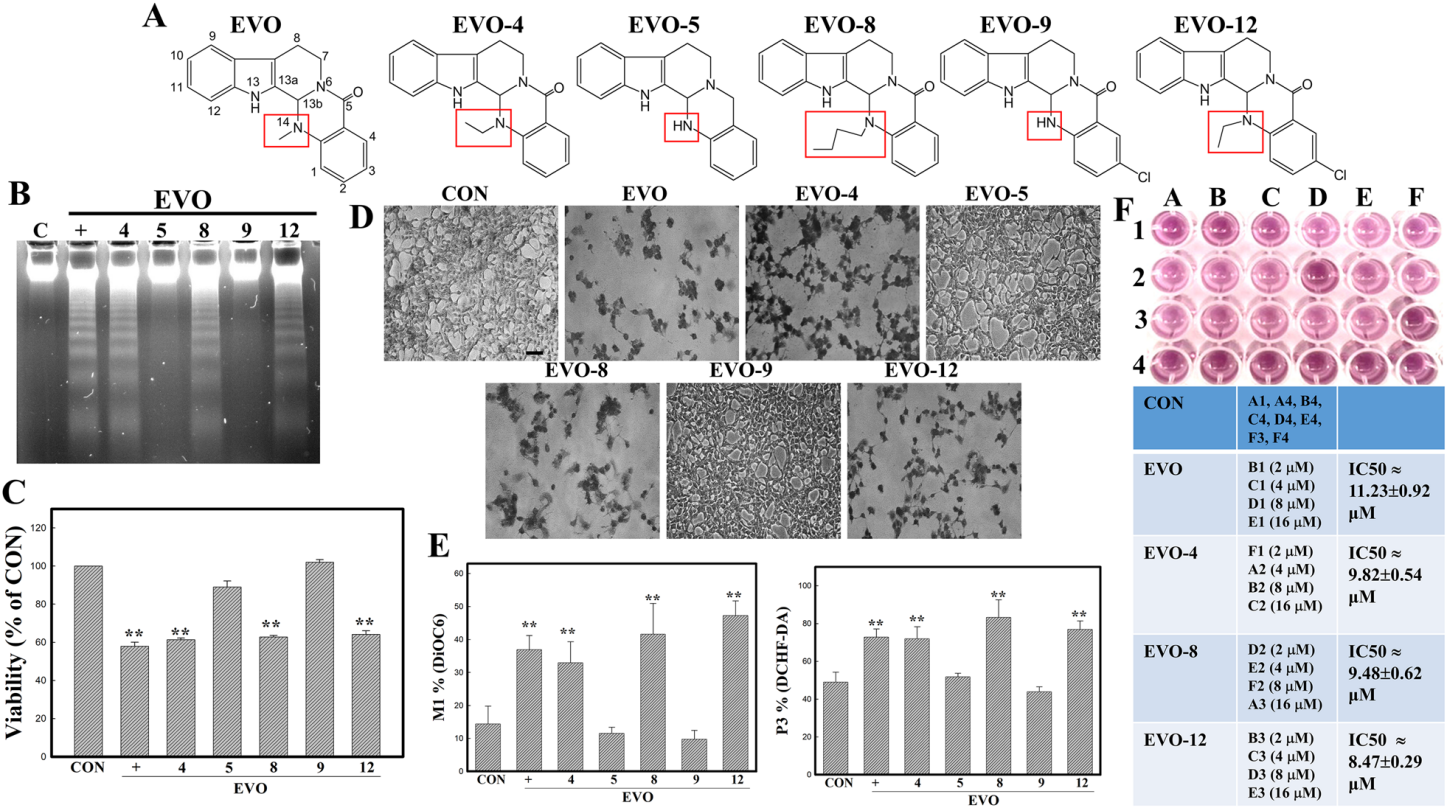
Structure-activity relationship of EVO and related chemicals on viability, DNA ladders, MMP, and ROS production in SW1736 cells. **A** The chemical structures of EVO and structurally related chemicals (EVO, -4, -5, -8, -9, and -12) are depicted. **B** Differential apoptotic effects elicited by EVOs in SW1736 cells. **C** Differential cytotoxicity of EVOs on the viability of SW1736 cells. **D** Differential morphological changes by EVOs in SW1736 cells. SW1736 cells were treated with the indicated EVOs (8 µM) for 12 h, and DNA integrity, viability, and morphology of SW1736 cells were analyzed by agarose electrophoresis, MTT assay, and Giemsa staining, respectively. Scale bar (), 100 μm. **E** Effects of EVOs on MMP and intraceullular peroxide production in SW1736 cells. SW1736 cells were treated with indicated EVOs for 12 h followed by detecting MMP and peroxide levels via DiOC6 and DCFH-DA staining via flow cytometric analysis. (F) IC50 values of EVO, EVO-4, -8, -12 on viability of SW1736 cells were examined by MTT assay. SW1736 were treated with different concentrations (2, 4, 8, and 16 µM) for 12 h, and viability of cells was measured by MTT assay. (Top) a representative of MTT assay; (Lower) IC50 values were measured via calculating the % cell viability and using equation by putting the 50 in the Y-value. Each data point was calculated from three triplicate groups, and data are displayed as the mean ± S.D. **p < 0.01 denotes a significant difference compared to the control (CON)

EVO and five structure-related compounds (EVO-4, 5, 8, 9, 12) were applied to study the structural importance of EVO on inducing apoptosis and p53 protein in SW1736 cells. Structures of these chemicals are depicted in Fig. 5A, and different substitutions at position 14 of EVO including methyl of EVO, an ethyl of EVO-4 and − 12, a hydrogen of EVO-5 and − 9, and a butyl of EVO-8 at position 14 are marked. Analysis of the DNA integrity in SW1736 cells treated with the indicated EVO-related chemicals showed that EVO, EVO-4, -8, and − 12 possessed the ability to induce DNA ladder formation in cells, whereas EVO − 5, and − 9 did not (Fig. 5B). Data of MTT assay showed that decreased viability of SW1736 cells by EVO, EVO-4, -8, and − 12 were observed (Fig. 5C). Additionally, EVO, EVO-4, -8, and -12 treatment reduced the cell number with increased chromatin condensed cells (arrow) via Giemsa staining under microscopic observations (Fig. 5D). Data of flow cytometric analysis of MMP and intracellular peroxide production by indicated EVOs showed that EVO, EVO-4,-8, -12 treatment disrupted the MMP and increased levels of intracellular peroxide in SW1736 cells using DiOC6 and DCFH-DA fluorescent dye, respectively (Fig. 5E). IC50 values of EVO and its analogs were examined via MTT assay. It indicated that IC50 values of EVO, EVO-4, -8, and − 12 are 11.23±0.92, 9.82±0.54, 9.48±0.62, and 8.47±0.29 µM, respectively (Fig. 5F). This implies that adding an alkyl group, such as a methyl or butyl, at position 14 of quinazolin is critical to apoptosis by EVOs in SW1736 cells, which cause loss of MMP and increase ROS production leading to apoptosis.

### Endogenous p53 status affects apoptosis and G2/M arrest in human ATC cells by microtubule disruptors paclitaxel, nocodazole, and EVO

**Fig. 6:**
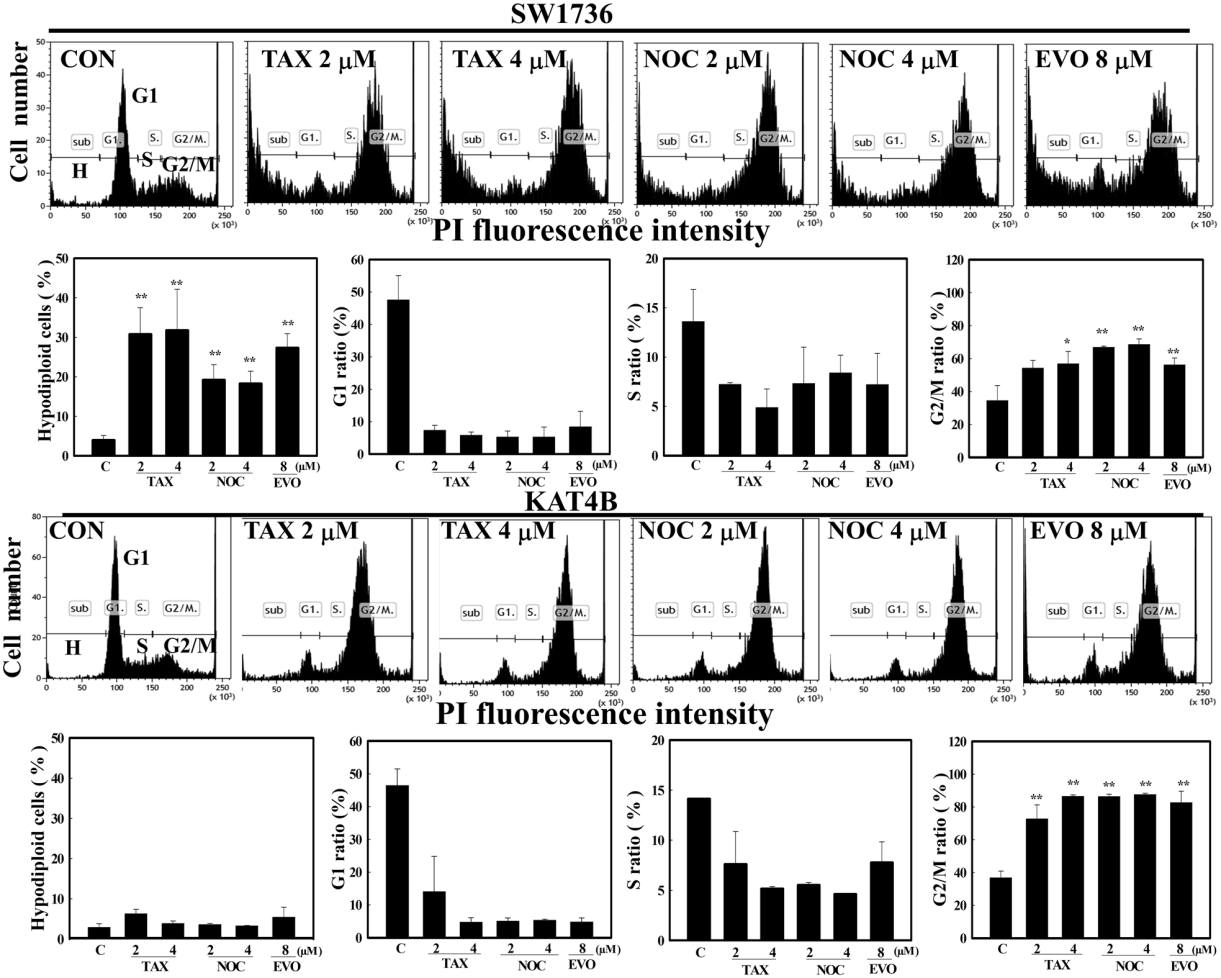
Differential actions of microtubule disruptors including EVO, paclitaxel (TAX), and nocodazole (NOC) on the ratio of hypodiploid cells, and ratio of cells in G1, S, and G2/M phase in ATC cells SW1736 (p53+/+) and KAT4B (p53 mut/mut) cells. SW1736 (Upper panel) and KAT-4B (Lower panel) cells were treated with indicated concentrations of EVO, TAX, and NOC for 24 h, and ratio of cells at sub-G1 (hypodiploid cells), G1, S, and G2/M phase was measured by flow cytometry via PI staining. A representative of the flow cytometric analysis was shown on the top of each panel; data were calculated from three triplicate groups and statistically analysis was shown below. Each data point was calculated from triplicate groups, and data are shown as the mean ± SD. *p < 0.05, **p < 0.01 denotes a significant difference from control group (C)

Our previous paper showed that EVO, paclitaxel (TAX), and nocodazole (NOC) were microtubule disruptors to inhibit growth of various cancer cells via increased tubulin polymerization [30]. We further examined if endogenous p53 status affect the sensitivity of human ATC cells to microtubule disruptors including TAX, NOC, and EVO leading to apoptosis or G2/M arrest by flow cytometric analysis. Data as illustrated in Fig. 6 showed that ratios of hypodiploid cells by these three chemicals in SW1736 cells were significant higher than those in KAT4B cells (Fig. 6). Similar pattern in ratio of G1 and S phase were observed in SW1736 and KAT4B cells by TAX, NOC, and EVO. Interestingly, the ratio of G2/M in KAT4B cells by TAX, NOC, and EVO was increased significantly, indicating KAT4B cells were preferentially arrested at G2/M phase under stimulations (Fig. 6). These results provide evidence to support that endogenous p53 status might affect the responses of human ATC cells to microtubule disruptors leading to apoptosis and G2/M arrest, respectively.

## Discussion

ATC is resistant to various treatments such as chemotherapy and radiation with minimal therapeutic effects on patients’ survival rates. The current study investigated the effects of EVO and the role of endogenous p53 status on apoptosis and cell cycle arrest of ATC cells. We found that EVO was able to reduce the viability of SW1736 and KAT4B ATC cells with increased apoptotic characteristics and an G2/M arrest ratio. Interestingly, SW1736 cells exhibited higher sensitivity to EVO-induced apoptosis than did KAT4B cells. An increase in the endogenous p53 protein was found in SW1736 cells but not in KAT4B cells, and treatment with the p53 inhibitor, pifithrin-α, and introducing p53 siRNA significantly inhibited EVO-induced apoptosis and G2/M arrest in SW1736 cells. A critical determinant of the endogenous p53 status by EVO’s actions in ATC cells was demonstrated herein.

p53 plays a critical role in either apoptosis or cell cycle arrest in response to various challenges, and cancer patients with wild-type p53 have better outcomes from chemotherapy than those with deleted or mutated p53 [31]. Both cell-cycle arrest and apoptosis are the most prominent outcomes of p53 activation, closely related to inhibit tumor development [32]. Emerging evidence supported that anti-tumor roles of p53 was mediated by regulating cellular processes including metabolism, antioxidant responses, and DNA repair [33]. Sriraman et al. (2018) reported that cell-cycle arrest by p53 is mediated by the transcriptional activation of p21/WAF to binding with cyclin E/Cdk2 and cyclin D/Cdk4 complexes which causes arrest at the G1 cell cycle [34]. Additionally, p53 activation also arrests cells at the G2/M phases via inhibiting cyclin B/Cdc2 and cell-cycle progression through mitosis [35]. Our previous reports in accordance with other papers indicated that EVO was shown to arrest cell cycle progression at the G2/M phase and induce apoptosis in various cancer cells including colon carcinoma, glioblastoma, and ovarian cancer cells, and the mechanism including activation of endoplasmic reticular (ER) stress and c-Jun N-terminal kinase (JNK) was identified [26, 30]. Although apoptosis and cell cycle modulation by EVO were reported, the role of the endogenous p53 status on the actions of EVO in ATC cells is still unclear. Pifithrin-α is a reversible p53 inhibitor, and apoptosis by UV irradiation, and doxorubicin, and paclitaxel treatment was inhibited by pifithrin-α, which was not seen in p53-null cells [36]. In the present study, we found that SW1736 cells (wtp53) showed higher sensitivity to EVO-induced apoptosis than did KAT4B cells (mutp53). Increases in the p53 protein and its associated proteins, including p21 and p27, by EVO were detected in EVO-treated SW1736 cells but not in KAT4B cells. The p53 inhibitor, pifithrin-α, and p53 siRNA significantly reversed the actions of EVO in SW1736 cells but not in KAT4B cells. This indicates that p53 is a determinant of sensitivity of ATC cells to EVO-induced apoptosis and cell cycle arrest.

Roles of mitochondria in chemical-induced apoptosis and cell cycle arrest of cancer cells were reported, and results are still controversial. For example, poncirin induced apoptosis in gastric cancer cells via an extrinsic apoptotic pathway [37], and quercetin induced mitochondrion-mediated apoptosis in U373MG cells [38]. In the presence of EVO stimulation, disruption of the MMP was observed in various cancer cells [24, 30]. Activation of Casp-3 to cause cleavage of the PARP protein located downstream of MMP disruption was identified in apoptosis, and both Casp-3-dependent and -independent apoptosis by EVO were observed in human leukemia and colon cells [39–41]. In the present study, EVO reduced the viability and induced apoptosis with decreased MMPs in ATC cells, and SW1736 cells showed higher disruption of MMPs by EVO than did KAT4B cells. This suggests that disruption of the MMP leading to mitochondrion-mediated activation of the Casp-3 cascade contributes to EVO-induced ATC cell apoptosis. Additionally, ROS are double-edged molecules for pro- and anti-apoptosis in response to several stimuli [42]. In the presence of EVO treatment, significantly increased intracellular peroxide levels were found in SW1736 cells, but the increase was less significant in KAT4B cells (Data not shown). Fang et al. [43] indicated that increased ROS production contributes to EVO-induced apoptosis of human small-cell lung cancer cells [43]. Ge et al. [44] showed that the antioxidative activity by EVO inhibits proliferation of vascular smooth muscle cells [44]. Both pro-oxidant and antioxidant effects of EVO were described in different cells, and this needs to be further studied.

We examined the structural importance for EVO-induced apoptosis of ATC cells here. Ogasawara and Suzuki (2004) also indicated the role of a methyl group at position 14 for EVO in inhibiting invasion by Lewis lung cancer and melanoma cells [45]. Our previous suggested that alkyl groups at position 14 is critical for apoptosis by EVO in various cancer cells including colorectal carcinoma, glioblastoma, and renal cell carcinoma cells [9]. As shown in Fig. 5, EVO, -4, -8, and − 12 containing a methyl, an ethyl or a butyl at position 14, showed significant apoptosis in ATC cells SW1736, compared with EVO-5 and EVO-9, both contains hydrogen group at position 14. The critical roles of alkyl substitutions at position 14 for inhibiting the viability of ATC cells via apoptosis by EVO were demonstrated.

## Conclusions

**Fig. 7:**
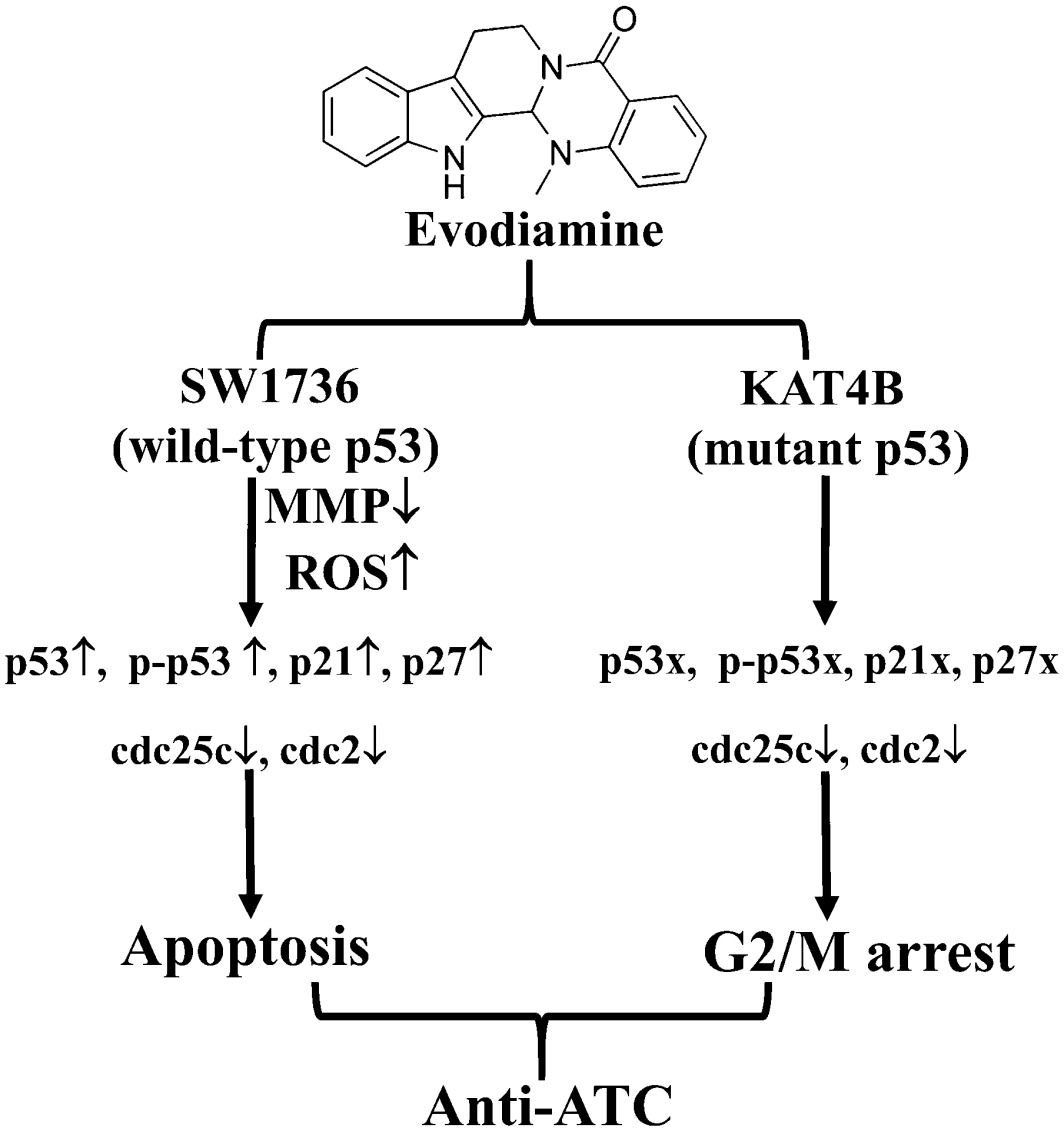
A tentative mechanism of p53’s action to induce apoptosis and G_2_/M arrest by evodiamine (EVO) leading to anti-anaplastic thyroid cancer (ATC) action is proposed herein. MMP, mitochondrial membrane potential. MMP, mitochondrial membrane potential; ROS, reactive oxygen species

This study demonstrated that the endogenous p53 status may affect the sensitivity of ATC cells to EVO-induced apoptosis and G2/M arrest. We showed that wild-type p53 SW1736 ATC cells expressed higher cell death and apoptotic cells than mutant p53 KAT4B ATC cells by EVO, and increased p53 and its downstream proteins, including p27 and p21, were detected in SW1736 cells but not in KAT4B cells. Incubation of both cell lines with the p53 inhibitor, pifithrin-α, or p53 siRNA reversed EVO-induced apoptosis and G2/M arrest in SW1736 cells but not in KAT4B cells. These findings provide evidence for a molecular signature of p53 in EVO’s effects in human ATC cells, and express the importance of p53 in antitumor actions of EVO against various human cancer cells. A tentative model indicating apoptosis for wtp53 ATC cell SW1736 and G2/M arrest for mutp53 ATC cell KAT4B by EVO was shown in Fig. 7, and that suggests the critical role of endogenous p53 status on suppression of ATC cell survival by microtubule disruptors such as EVO, paclitaxel, and nocodazole.

## Data Availability

The datasets used and/or analysed during the current study are available from the corresponding author on reasonable request.

## Abbreviations

α-TUB: α-Tubulin
ANOVA: Analysis of variance
EVO: Evodiamine
Wtp53: Wild-type p53
Mutp53: Mutant-type p53
ATC: Anaplastic thyroid cancer
BCIP: 5-Bromo-4-chloro-3-indole phosphate
Casp: Caspase
FBS: Fetal bovine serum
MMP: Mitochondrial membrane potential
JNK: c-Jun N-terminal kinase
MTT: 3-(4,5,-Dimethylthiazol)-2-yl-2,5-diphenyltetrazolium bromide
NBT: Nitroblue tetrazolium
PI: Propidium iodide
PARP: Poly(ADP-ribose) polymerase
ROS: Reactive oxygen species
SDS: Sodium dodecylsulfate
SD: Standard deviation
DCFH-DA: 2′,7′-Dichlorodihydrofluorescein diacetate
